# A Panoramic View of Narrow Band Imaging in the Treatment of Head and Neck Cancer

**DOI:** 10.2174/0115734056405507251121110733

**Published:** 2025-11-28

**Authors:** Xiang Zheng, Quansheng Fu, Jie Qian, Li Li

**Affiliations:** 1 Department of Otorhinolaryngology Head and Neck Surgery, Wuxi Branch of Ruijin Hospital, Shanghai Jiao Tong University School of Medicine, Wuxi, China

**Keywords:** Narrow band imaging, Head and neck cancer, Traditional white-light imaging, Computed tomography, Malignant tumors, Magnetic resonance imaging

## Abstract

**Introduction:**

This study aimed to systematically review the application of narrow band imaging (NBI) in the diagnosis, treatment, and follow-up of head and neck cancer.

**Methods:**

Through literature review and generalization of our clinical experiences, this review thoroughly described the features, mechanisms, advantages, drawbacks, and prospects of NBI in the treatment of head and neck cancer.

**Results:**

NBI is an emerging endoscopic technology that emits an ambient light at wavelengths of 415 nm (blue) and 540 nm (green) to clearly visualize the details on the mucosal surface. It presents potent efficiencies in the preoperative, intraoperative, and postoperative surveillance and diagnosis of head and neck cancer.

**Conclusion:**

NBI is a front-edge imaging technology that allows early screening, precise treatment, and postoperative monitoring of head and neck cancer.

## 1 INTRODUCTION

Head and neck cancer is prone to metastasis and infiltration into adjacent organs, leading to high mortality and a poor quality of life. Therefore, early detection, diagnosis, and intervention carry a high prognostic value. Endoscopy has proven effective in recognizing head and neck cancer. However, traditional white-light imaging (WLI) is quite limited in the early detection of submucosal lesions.

Narrow-band imaging (NBI) has emerged as a new endoscopic technology for cancer diagnosis. In the NBI system, the endoscopic light source is filtered out, only leaving an ambient light at wavelengths of 415 nm (blue) and 540 nm (green) that can clearly visualize the details on the mucosal surface. Here, we review the performance of NBI in the preoperative, intraoperative, and postoperative surveillance and diagnosis of head and neck cancer.

This literature review first systematically searched for rele-vant articles on NBI in head and neck cancer published over the past two decades, with particular emphasis on studies from the last five years. Two investigators independently performed the literature search in PubMed, manually reviewed citations, and extracted the relevant data. Briefly, sections descr-ibing the development of NBI and its application to the diagnosis, treatment, and follow-up of head and neck cancer were thor-oughly reviewed. Finally, we have outlined the limitations and future innovations of NBI in the treatment of head and neck cancer, based on a literature review and our clinical experience.

## 2 FEATURES OF NBI

### 2.1 Development of NBI

As a smart technology, NBI was co-developed by Olympus Medical Systems (Tokyo, Japan) and the National Cancer Center East Hospital in 1997, and initially applied to diagnose digestive diseases in 2021 [1]. In 2006, the EVIS EXERA II and EVIS LUCERA SPECTRUM endoscopic video systems, including NBI, were introduced into Japan. The NBI system was later introduced into China in 2008.

The NBI system contains a monitor, a light source unit, optical filters, and components that are essentially the same as those in the red-green-blue sequential video-endoscope (*e.g*., a monochromatic charge-coupled device and an imaging processor) [2, 3]. Despite its widespread use, the first-generation NBI has shown limitations in diagnosis, including insufficient brightness and annoying noise during imaging [4]. To resolve them, the second-generation NBI has emerged, with a high-intensity discharge lamp added to intensify the brightness in the light source. Compared to the first-generation, it offers a detectable distance twice as long and a view that is brighter and clearer [5].

### 2.2 Optical Mechanism of NBI

In traditional electronic endoscopes, xenon lamps provide a source of white light, composed of three colors at wavelengths of 605 nm (red), 540 nm (green), and 415 nm (blue) [6]. NBI captures information that varies with the depth of penetration and the wavelength of light [7]. The absorption and scattering of light in human tissues exhibit different patterns [8]. Generally, light with a shorter wavelength penetrates more shallowly, while that with a longer wavelength penetrates deeper. Accordingly, a special optical filter is anchored to the NBI system, allowing a narrowband of just two colors: blue light (415±15 nm) and green light (540±15 nm) [9]. Penetrating by about 100-200 μm in depth, the blue light is mainly absorbed by the hemoglobin in the capillaries of the mucosa and thus displayed as brown. The green light can penetrate deeper into the submucosa to a depth of 300-500 μm and is absorbed by hemoglobin in the deeply situated blood vessels; thus, the green light appears as cyan [10, 11].

Blood vessels show color variations with depth under NBI. Either brown (mucosal surface capillary network) or cyan (deep venous network) endows a clear image of vascular morphology and distribution. This ability makes NBI a valuable imaging tool for medical diagnosis [12]. WLI absorbs the entire visible spectrum (400-700 nm), making images less reliable for contrast-enhanced blood vessel visualization. From the perspective of physics, NBI is an optimization of WLI with a more stringent spectral screening [13].

### 2.3 Contrast and Resolution of NBI

The selection of blue and green light is not arbitrary but is based on the spectral absorption properties of hemoglobin [14, 15]. The absorption spectrum of hemoglobin shows two peaks at 415 nm and 540 nm [16, 17]. Therefore, NBI achieves a high contrast and resolution of blood vessels. First, this high contrast enables visualization of the microvascular network, including tiny capillaries with diameters of only 5-10 μm [18, 19]. The resolution achieved with NBI far exceeds that of traditional endoscopic techniques [20]. Ota *et al*. confirmed the diagnostic accuracy of NBI in high-grade intraepithelial papillary capillary loops (IPCLs) [21]. NBI displays a capillary pattern of the subepithelial layer surrounding the crypt opening of duodenal neoplasms, but this pattern is hardly revealed by a conventional endoscopy [22]. Second, NBI can simultaneously manifest tissue structures at different depths, providing hierarchical vascular information [23, 24]. NBI at depths of 240 µm, 200 µm, and 170 µm enables observation of layered structures and superficial lesions [25]. Yoshida *et al*. made a diagnosis of esophageal cancer based on the invasion depth on NBI images, obtaining a 16.7% higher accuracy rate than the conventional magnifying endoscopy [26]. Third, NBI can selectively target tumor lesions, especially premalignant or malignant lesions rich in neovascularization [24, 27, 28]. In comparison to the conventional WLI alone, NBI combined with cystoscopy increases the detection rate of recurrent bladder cancer by 24.1% [29]. Moreover, the sensitivity of NBI in diagnosing bladder carcinoma *in situ* is significantly higher than that of WLI (100% *vs*. 83%). Finally, NBI can be operated simply and conveniently with a single button switch from the normal white light mode to NBI mode. Without the need for additional contrast agent, NBI reduces patient discomfort and the risk of events related to contrast injection [30].

NBI offers unique advantages compared to other endoscopic techniques (Table **1**). WLI, already established as a mainstream tool in neoplasia diagnosis, shows a high accuracy in brightly displaying delicate structures, but not the contents underneath the tissue surface [31]. Lugol chromoendoscopy is effective for screening early-stage esophageal cancer, and as a navigator in complex surgeries, it ensures a complete resection of tumor lesions and reduces the risk of postoperative recurrence. It also provides real-time intraoperative guidance for the removal of malignant lesions. However, Lugol chromoendoscopy takes a long time and causes adverse events, like iodine allergy, heartache, and nausea [32]. Compared to Lugol chromoendoscopy, NBI is a virtual method without the need for spraying pigments, thus avoiding uneven pigment distribution [33], inhalation of Lugol solution, or allergic reactions [34].

Being non-invasive, high-resolution, and optical and digital, both NBI and autofluorescence imaging (AFI) are advantageous over WLI in many respects. However, AFI is inferior to NBI in identifying tumor lesions with a thickened mucosal layer, increased blood flow, and decreased collagen fluorophores in the submucosal layer [35]. Ni *et al*. reported significantly higher specificity (85% *vs*. 48%) and accuracy (91% *vs*. 77%) of NBI in diagnosing laryngopharyngeal tumors than AFI [36]. Confocal laser endomicroscopy (CLE), combining the features of confocal microscopy and endoscopy, can achieve real-time imaging at the microscopic level and high-resolution visualization at the subcellular level, but is challenged by complications related to fluorescent agents, difficulties in manipulating tiny machine parts, and integrating them into endoscopic devices [37]. Compared to CLE, NBI employs simpler devices and is easier to operate. In addition, CLE is not so accurate as NBI in distinguishing colorectal lesions (66.7% *vs*. 71.9%) [38]. All these advantages of NBI contribute to its wide applications in clinical settings.

### 2.4 System Calibration, Sensitivity Thresholds, and Imaging Processing of NBI

System calibration is the cornerstone of NBI for precisely capturing and interpreting light within specific wavelength bands, typically involving sensor characterization, radiometric calibration, and the elimination of artifacts and stray light. It is particularly well-suited for clearly visualizing blood vessels and mucosal surfaces in complex anatomical regions, such as the head and neck. Currently, star clusters in the Sloan Digital Sky Survey (SDSS) are frequently used to calibrate NBI images [39]. Sensitivity thresholds vary in targeted regions. A meta-analysis established the sensitivity and specificity of NBI, especially in distinguishing malignant head and neck cancers from benign lesions [40]. By analyzing 6,187 lesions across 25 articles, it was concluded that NBI's sensitivity for diagnosing head and neck cancer can reach 88.5%. Both enhancement algorithms and segmentation tools are common methods to process NBI images. The former, aimed at adjusting for contrast, reducing noise, and extracting critical features, seems more favorable for the diagnosis and classification of head and neck cancer, which is characterized by subtle anatomic structures. Fang *et al*. trained NBI images using the YOLOv5 algorithm, achieving the goal of accurately diagnosing esophageal carcinoma [41]. Automatic segmentation algorithms are more frequently used to examine gastrointestinal lesions with segmental features [42].

## 3 CLINICAL APPLICATION OF NBI IN HEAD AND NECK CANCER

### 3.1 Head and Neck Cancer

Head and neck cancer is a group of malignant tumors located in the head and neck region (excluding the brain), including oral, nasopharyngeal, oropharyngeal, hypopha-ryngeal, laryngeal, sinus, salivary gland, and thyroid cancers. Alcohol and tobacco consumption are the dominant causes of head and neck cancer [43, 44]. Other established risk factors consist of betel nut eating, human papillomavirus (HPV) infection, radiation exposure, and exposure to toxins [45]. Globally, head and neck cancer is the seventh most common cancer and the ninth most common cause of death [46], accounting for more than 660,000 new cases and 325,000 deaths annually [47]. Its incidence reaches 3% in the United States and is even higher in older adults aged 55-65 years [48]. In developed countries, the average 5-year survival rate is 68.5%, which varies with tumor stage [44].

Computed tomography (CT) and magnetic resonance imaging (MRI) are the mainstream diagnostic tools for head and neck cancer [49]. On CT images, head and neck cancer is characterized by reduced soft-tissue density, blurred margins, invasion into surrounding tissues, and vascular changes (*e.g*., increased numbers of blood vessels, irregular blood vessel courses, and thickened, curved lumens) [50, 51]. MRI provides high contrast of soft tissues and allows evaluation of the location, infiltration, spread, and stage of head and neck cancer [52, 53]. Moreover, MRI is a common tool to monitor the response to treatments during the follow-up visits.

However, the accuracy of both imaging techniques is unsatisfactory due to the complex anatomy of small-volume lesions in the head and neck. It has been proven that both CT and MRI are less reliable in clearly displaying premalignant or malignant head and neck cancer enriched with vascular networks [54] (Table **2**).

### 3.2 Preoperative Evaluation with NBI for Head and Neck Cancer

For preoperative evaluation, traditional imaging tools have methodological limitations in identifying early and superficial head and neck cancer lesions. For example, CT is limited by the interference from personal artifacts and the extensive exposure to ionizing radiation. Metal artifacts, such as dental restorations, mainly affect tumor detection in the head and neck areas, especially the root of the tongue and palate [55]. These false features on CT images may cause misdiagnosis or delayed diagnosis.

In NBI, the blue light (wavelength 415 nm) can penetrate the mucosa to the superficial vasculature in the head and neck, thereby identifying superficial mucosal lesions that are hardly detected by WLI. Therefore, NBI shows high value not only for early screening of head and neck cancer but also for determining its scope and type. Muto *et al*. screened superficial lesions in the oropharynx and hypopharynx of oesophageal cancer patients using NBI [56], showing “brown spots” as a feature of oral squamous cell carcinoma (OSCC), which is histologically confirmed as dilated capillaries by immunohistochemical staining [57, 58]. This microvascular hyperplasia pattern (MVP) can be observed by NBI in all tumor lesions but is rarely detected by conventional imaging tools, even with enhanced imaging [59]. Given that angiogenesis is a typical feature of carcinogenesis, NBI is thus emphasized for its clinical significance in identifying head and neck cancer in an early stage.

In a cohort of 217 patients with oesophageal cancer, 4 superficial lesions with a size of 5 cm or below were successfully identified by NBI, rather than conventional endoscopy. Consistently, scattered brown spots are also observed in tumor lesions [60]. In addition to NBI's higher sensitivity than endoscopy for detecting small superficial tumor lesions in the oropharynx and hypopharynx, the NBI system's small size allows thorough detection through the nasal cavity or convenient screening at the outpatient department. Among the 62 patients with 91 vocal cord leukoplakia, NBI effectively recognized malignant lesions with a sensitivity of 100%, a specificity of 97.4%, and an accuracy of 97.8% [61].

Based on the preoperative NBI manifestations of mucosal IPCLs, laryngeal lesions are categorized into five types, with type I-IV lesions considered as benign, and type Va-Vc lesions as malignant (Fig. **1**). Using the Ni classification, NBI screened out malignant laryngeal lesions from a total of 104 lesions with acceptable sensitivity and specificity (sensitivity, 100% in type Va and 83.8% in type Vb-Vc; specificity, 79.5% in type Va and 100% in type Vb-Vc) [62]. In a retrospective study involving 63 patients with chronic non-healing oral ulcers, NBI and IPCL classification could sensitively (93.75%) and specifically (91.49%) identify pre-malignant and malignant OSCC [63]. Early and precise detection of oral malignancies with NBI not only reduces mortality but also saves medical costs and time for pathological confirmation. Among the 96 patients with 206 suspected premalignant laryngeal and pharyngeal lesions, the vast majority of lesions (88%) could be accurately detected by NBI combined with WLI [64]. Notably, there is a significant correlation between the Ni classification and histological stage.

In addition to the classification of IPCLs, NBI provides sound evidence to classify vascular patterns in the oral cavity (Fig. **2**) [65]. Piazza *et al*. compared the diagnostic performance of NBI, conventional oral examination (COE), and WLI in oral and oropharyngeal subsites [66]. With the excisional biopsy and pathology as the gold standard, NBI significantly decreased the false positive rate (15% *vs*. 27% *vs*. 32%) and false negative rate (11% *vs*. 22% *vs*. 49%), compared to COE and WLI, in the classification of vascular patterns in the oral cavity. Therefore, preoperative NBI can provide precise vascular features that favor the formulation of an individualized surgical plan for head and neck cancer.

### 3.3 Intraoperative Application of NBI in Head and Neck Cancer

A tumor cell-free surgical margin can strongly indicate a good prognosis of head and neck cancer [67], while a negative margin ensures a low local recurrence rate and an all-cause mortality in patients [68, 69]. However, a negative margin is particularly difficult to delineate due to the infiltration into important structures, irregular tumor morphologies, and hidden or undetectable tumor cells. The incidence of positive margins in head and neck cancer surgeries remains high, ranging from 10% to 30% [70]. Intraoperative NBI can tailor a navigational surgical resection by defining the tumor scope and the surgical margin. Meanwhile, a minimal curative resection guided by intraoperative NBI can better preserve the functions of organs.

Klimza *et al*. compared the performance of intraoperative WLI and NBI in identifying early and moderately advanced laryngeal cancer [71]. Among the 90 suspected samples, NBI gained higher sensitivity (100% *vs*. 79.5%) and accuracy (85.7% *vs*. 71.1%) in confirming carcinoma *in situ* or invasive carcinoma than WLI. Attributed to the NBI-guided classification of IPCLs, tumor lesions can be distinguished from dysplastic lesions intraoperatively. In a pilot study involving 16 patients with oral and oropharyngeal carcinomas, the resection margin was initially set at 1.5 cm away from the macroscopic tumor, and intraoperative NBI was used to guide the resection. At the margins initially defined, NBI showed a positivity rate of 75%, suggesting that NBI could define a larger positive area to improve the prognosis and prevent postoperative recurrence [72]. NBI allowed a 240-µm penetration, thus favoring the intraoperative assessment of OSCC arising from thin mucosa (*e.g*., lateral tongue, buccal mucosa) [73]. A prospective study demonstrated intraoperative NBI to increase the R_0_ resection rate in patients with malignant lesions in the oral cavity and pharyngolaryngeal carcinomas, thus obtaining more histologically negative margins according to the conventional 15-mm definition [74]. Overall, a portable NBI system guides surgeons to dynamically judge the margin of head and neck cancer throughout the surgery, thus significantly improving the accuracy of surgical removal.

The combination of NBI and microsurgery systems has transformed the surgery for head and neck cancer into a more minimally invasive and precise procedure. By integrating NBI with surgical microscopy or endoscopic microscopy, precision resection can be achieved by clarifying changes in microvascular structures under high-power magnification [75, 76]. This combination has shown significant benefits in minimally invasive vocal cord surgery, transnasal endoscopic surgery, and robot-assisted surgery. In minimally invasive vocal cord surgery, NBI-oriented enhanced microscopy increases the success rate of surgery. In the treatment of early glottic cancer, the goal is to completely remove the tumor and preserve maximal normal vocal cord tissue and function. A single microsurgery is not accurate enough to judge the boundaries of tiny lesions, especially superficial lesions, like vocal fold leukoplakia and dysplasia. Transoral laser microsurgery (TLM), as a common minimally invasive surgery, can remove head and neck cancer lesions that are not suitable for robotic surgery, without damaging the normal tissue. Garofolo *et al*. assessed the rate of positive superficial margins in 82 early glottic cancer patients treated with TLM and its combination with intraoperative NBI [77]. The combination significantly decreased the rate of positive superficial margins (3.6% *vs*. 23.7%).

In summary, NBI has been found to be beneficial in either preoperative evaluation or intraoperative identification of positive margins during TLM for resecting laryngeal cancer [78].

### 3.4 Postoperative Evaluation with NBI for Head and Neck Cancer

The value of NBI has been increasingly recognized in postoperative evaluation of recurrences, treatment response, individualized adjuvant treatments, and secondary primary malignancy (SPM). In postoperative follow-up, an early detection of recurrent head and neck cancer is closely associated with a good prognosis.

Chu *et al*. highlighted the value of NBI in postoperative follow-up of OSCC patients [79]. Earlier than conventional tools, NBI detected more second primary malignant OSCCs (18% *vs*. 9%) at lower stages (4% *vs*. 37%), and in the NBI-monitored group, the proportion of patients requiring adjuvant therapy (12% *vs*. 50%) was lower. In a follow-up study of 66 patients with larynx and hypopharynx cancers initially treated with radiotherapy or chemoradiotherapy, post-treatment NBI sensitively and specifically identified 79% of suspicious recurrences that were later histologically determined to be malignancies [80]. Notably, 3 of the 19 recurrent malignancies were detected by NBI within 6 months of follow-up. A meta-analysis showed higher sensitivity (95% *vs*. 72%), specificity (96% *vs*. 72%), and diagnostic odds ratio (DOR) [433 (95%CI 120-1, 560) *vs*. 7 (95%CI 0-191)] of NBI, compared to WLI, in the follow-up examination for head and neck cancer [81]. Postoperatively, NBI detected 13 recurrent tumors and 2 metachronous tumors among 17 suspicious glottic lesions after surgical treatment of laryngeal squamous cell carcinoma (LSCC) [82]. NBI can also be used to individualize adjuvant therapy. For example, adjuvant radiotherapy is commonly recommended to patients with a negative margin within 5 mm, and may cause overtreatment, but after accurate evaluation with NBI, unnecessary radiotherapy can be avoided.

## 4 NBI BENEFITS PATIENTS WITH HEAD AND NECK CANCER

The narrowband spectrum of NBI is conducive to enhancing the visualization of mucosal blood vessels. NBI is superior to traditional endoscopies in displaying tumor lesions accompanied by microvascular changes. At present, NBI has greatly improved the diagnostic and detection rates of esophageal squamous cell carcinoma (ESCC), Barrett's esophagus, early gastric cancer, and early colon cancer [83-86].

The head and neck region harbors important organs with complex anatomical relationships and high heterogeneity. Similar to traditional endoscopy, NBI is a safe, time- and cost-saving tool without exposure to radiation [87]. In the early stage of head and neck cancer, NBI can detect premaligna-ncies/malignancies by visualizing tiny, brown blood vessels in the mucosa [88]. In sharp contrast with the surrounding mucosa shown in green, NBI effectively distinguishes head and neck cancer lesions from normal tissues, with an accuracy higher than conventional endoscopic techniques and equal to that of the gold standard of pathological analysis.

### 4.1 NBI can Increase the Diagnostic Accuracy of Head and Neck Cancer

In a meta-analysis involving 169 patients, NBI exhibited a higher pooled DOR (82.15, 99% CI, 7.06-955) and overall detection rate (0.35, 99% CI, 0.18-0.53) for head and neck squamous cell carcinoma of unknown primary origins, indicating the indispensable edge of NBI in pinpointing the origin of head and neck cancer [89]. The area under the curve (AUC) of NBI was up to 96.94% in distinguishing head and neck cancer out of 6,187 suspicious lesions [40]. In comparison to WLI, NBI resulted in a higher detection rate of oral (100% *vs*. 92%), oropharyngeal (100% *vs*. 69%), hypopharyngeal (100% *vs*. 38%), and laryngeal malignancies (100% *vs*. 37%), although the detection rates were comparable for T2-T4 head and neck cancer [90]. NBI has been suggested to be superior to conventional imaging techniques for detecting early-stage head and neck cancer. During differentiation from dysplasia (mild, moderate, and severe) to neoplasia, NBI has been found to be highly reliable for identifying premalignant and malignant lesions in mice with 4-NQO-induced oral cavity cancers, with diagnostic accuracy ranging from 86% to 98% [91].

### 4.2 NBI can Facilitate Targeted Biopsies for Head and Neck Cancer

An accurate preoperative assessment of head and neck cancer avoids unnecessary punctures and biopsies, which are painful, invasive, and harmful to the quality of life. Common complications for these procedures include bleeding, infection, and nerve injury. NBI-guided targeted biopsies are characterized by accuracy and convenience, especially in superficial lesions in the head and neck. Yang *et al*. reported the higher accuracy of NBI-targeted biopsies for laryngeal lesions (95.6% *vs*. 75.7%) and superficial flat laryngeal lesions (89.3% *vs*. 41.4%) than routine biopsies [92]. Additionally, NBI-guided targeted biopsies are much cheaper, thus reducing the financial burden of patients [93].

### 4.3 NBI can Detect Field Cancerization in Head and Neck Cancer

Head and neck cancer is highly likely to recur and metastasize, or induce SPM that causes a 2-fold mortality higher than that of distal metastases [94]. Traditional imaging techniques are limited in recognizing SPM of head and neck cancer. Generally, the high incidence of SPM in patients with head and neck cancer is attributed to field cancerization, that is, multiple early lesions or premalignancies are independently developed around the primary head and neck cancer. During the surgical resection of head and neck cancer, field cancerization is often observed at the edge of the resection site, leaving the “tumor spark” in the body. NBI can navigate the complete resection of multifocal lesions. Concurrent ESCC is a complication that affects 10%-20% of patients with head and neck cancer and leads to a poor prognosis. However, traditional endoscopy has a limited ability to detect superficial or precancerous lesions. In a cohort of 21 patients with esophageal neoplasia, the majority (76.2%) suffered from synchronous lesions, and NBI successfully recognized both dysplasia and neoplasia [95]. Nonaka *et al*. reported a consecutive series of 424 ESCC patients monitored by NBI after chemoradiotherapy or surgery [96]. NBI showed its superiority in identifying metachronous superficial pharyngeal cancers than conventional endoscopy (10.9% *vs*. 1.2%).

### 4.4 NBI can Allow an Efficient Surveillance of Head and Neck Cancer

An efficient post-treatment surveillance is expected to decrease the recurrence and prolong the long-term survival [97]. Existing guidelines recommend a follow-up examination every 1-3 months in the first year, 2-6 months in the second year, and 4-8 months afterward. NBI can be easily performed in outpatient settings, making it ideal for follow-up surveillance.

Piazza *et al*. conducted a surveillance study involving a cohort of 390 patients with head and neck squamous cell cancer having been treated with radiotherapy and/or chemotherapy [98]. NBI, combined with a high-definition television camera, exhibited a capability of catching persistent, early recurrent, and metachronous tumors, with an accuracy of 92%, a sensitivity of 100%, and a specificity of 98%. Kanaan *et al*. highlighted the potential of NBI as a routine surveillance examination following the surgery for sinonasal malignancies [99]. They reported that two patients with sinonasal malignant melanomas who were regularly followed up with NBI did not present recurrence at 22 and 24 months postoperatively, indicating that NBI is a safe and effective tool for surveillance of the postoperative condition of patients.

## 5 DRAWBACKS OF NBI

Despite the advantages of NBI, its drawbacks should be considered. First, the quality of NBI images is associated with light source, filters, and sensors. Due to a low transmittance into the tissue, a sufficient light source is required for the NBI system to obtain a high-quality image in the head and neck regions. Second, NBI is inferior to traditional imaging techniques for visualizing deep tissues. Third, unmagnified NBI images are usually blurry, failing to show the structure and color changes in lesions situated within larger lumens. Fourth, the steep learning curve of NBI may increase the false-positive rate, leading to unnecessary biopsies [100, 101]. A total of 134 NBI records are used to generate a learning curve for diagnosing laryngeal cancer, with the histological diagnosis as the gold standard. Generally, 65-70 NBI examinations are needed to reach the plateau of the learning curve. NBI performed by inexperienced endoscopists has been found to have lower sensitivity than dye-spray chromoendoscopy for screening high-grade esophageal squamous mucosal neoplasia compared to experienced endoscopists (53% *vs*. 100%) [102]. Fifth, NBI has to be combined with other imaging examinations (*e.g*., PET, CT) in special cases of head and neck cancer. Sixth, the price of the NBI system is still high for some primary healthcare institutions in underdeveloped regions [103].

## 6 FUTURE PROSPECTS

Artificial intelligence (AI), especially deep learning technology, has shown great potential in magnifying the performance of medical imaging tools [104]. AI-assisted NBI can overcome the limitations of subjective judgment by endoscopists [105]. An AI-based convolutional neural network (CNN) through deep learning algorithms was trained using NBI images from 1,039 LSCC patients and 1,388 patients with benign lesions in the larynx. The performance of AI-based CNN was surprisingly comparable to that determined by experienced otolaryngologists (accuracy 90.91% *vs*. 91.93%±3.20%) [106]. Massive, complex, private data from NBI images challenge precise interpretation and diagnosis, which can be skillfully addressed by AI-driven large language models [107, 108]. While AI assistance may accelerate image acquisition and reduce labor costs, it cannot yet be said to have fully replaced humans in interpreting information from NBI images.

In addition, it is necessary to construct a multimodal imaging platform involving NBI and other imaging technologies, such as fluorescence imaging, CLE, and optical coherence tomography, to simultaneously provide anatomical, functional, and molecular information of tissues for better surveillance, monitoring, and treatment. Currently, as the core component of the NBI system, the narrowband filtering system should be upgraded in the future. Tunable filter systems, multi-band filter technology, nanomaterial filters, and AI-based light source control systems deserve in-depth exploration.

Besides, portable and cheaper NBI systems should be developed for primary medical institutions, allowing a large-scale triage for high-risk individuals of head and neck cancer. Palliative NBI should also be designed to carry out targeted biopsies for those with advanced head and neck cancer in the future.

## CONCLUSION

NBI is an emerging endoscopic technology that stands at the frontier of cancer imaging. Only depending on an ambient light at wavelengths of 415 nm (blue) and 540 nm (green), it has significantly improved preoperative screening, intraoperative precise treatment, and postoperative monitoring of head and neck cancer. In particular, NBI can increase the diagnostic accuracy of head and neck cancer through a clear vascular classification. By defining a negative margin, NBI facilitates targeted biopsies and the detection of field cancerization in head and neck cancer. Moreover, NBI is effective, convenient, and cost-friendly for postoperative follow-up of head and neck cancer, thus minimizing the risk of recurrence. Nevertheless, NBI remains to be modified to benefit a larger population. Its integration with the latest AI techniques can assist in real-time diagnostics of head and neck cancer and other cancers, and its economically friendly design is also expected to lower medical costs.

## Figures and Tables

**Fig. (1):**
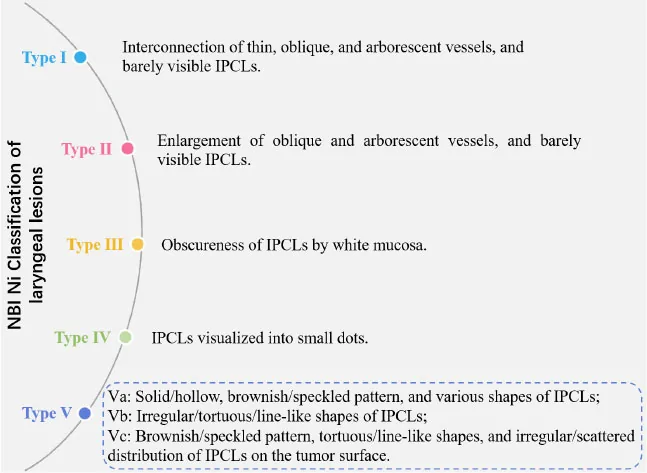
Ni classification of laryngeal lesions based on the patterns of mucosal IPCLs.

**Fig. (2):**
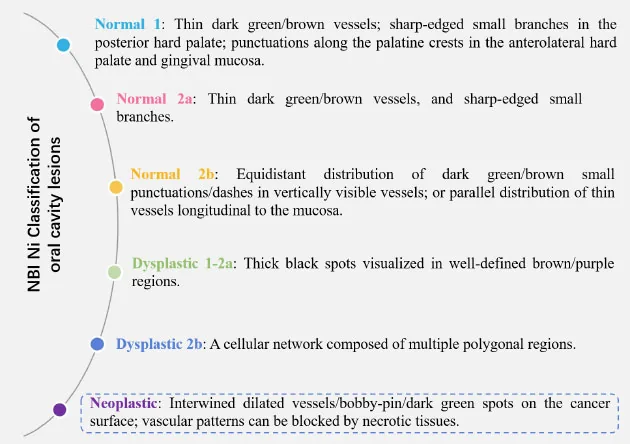
Ni classification of oral cavity lesions based on the vascular patterns.

**Table 1:** Pros and cons of common endoscopic techniques.

**Endoscopic Techniques**	**Pros**	**Cons**
WLI	· High diagnostic efficiency for neoplasia · Brightness	· Not ideal for displaying the mucosal surface · Less specific and sensitive in detecting neoplasia
Lugol chromoendoscopy	· Surgical navigation · Complete resection · Real-time intraoperative guidance	· Surgery prolongation · Iodine allergy · Adverse events
AFI	· Non-invasiveness · High resolution	· Inferior in identifying tumor lesions with thickening of the mucosal layer, increased blood flow, and decreased collagen fluorophores in the submucosal layer
CLE	· Real-time tissue imaging at the microscopic level · High-resolution visualization at the subcellular resolution	· Complications related to the fluorescent agent · Difficulties in manipulating tiny imaging equipment and integrating them into endoscopic devices
NBI	· Maximum contrast of vessels and the surrounding mucosa · Classification of vascular patterns for guiding the surgery and treatment	· The need for considerable training · Low brightness · High cost of the NBI system

**Abbreviations**: WLI, white light imaging; AFI, autofluorescence imaging; CLE, confocal laser endomicroscopy; NBI, narrow band imaging.

**Table 2:** A summary of detailed demographics, anatomical regions, image acquisition protocols, or scanner models of NBI in detecting head and neck cancers.

**Cancer Type**	**Anatomical Regions**	**Sample Size**	**Patient Demographics**	**NBI Mode**	**Reference**
SCC *in situ*	Oropharyngeal and hypopharyngeal mucosal sites	34 consecutive superficial lesions in 18 patients	Age: 59.5 years (range, 43-71 years) Sex ratio: 4.9:1	F1 filter [center wavelength, 415 nm; full width at half-maximum (FWHM), 30 nm] to the B channel, the F2 filter (center wavelength, 445 nm; FWHM, 30 nm) to the G channel, and the F3 filter (center wavelength, 500 nm; FWHM, 30 nm) to the R channel	[51]
SCC *in situ*	Esophagus	217 patients	Age: 66.7±7.9 years Sex ratio: 7.0:1	Two-band color-chip NBI system [video system center CV-160B, light source (Xenon-lamp) CLV-160B, and rhinolaryngo-videoscope VISERA ENF-V2]	[55]
High-grade dysplasia, carcinoma *in situ*, and invasive cancer	Vocal folds	91 leukoplakia changes on vocal folds in 62 patients	Sex ratio: 2.9:1	Visera Elite OTV-S190 video system and endoscopic nasopharyngolaryngoscopy-VH videoendoscope (Olympus Medical Systems, Volketswil, Switzerland)	[56]
Carcinoma *in situ* and invasive cancer	Laryngeal region	85 patients	Age: 55 years (range, 36–83 years) Sex ratio: 16:1	Olympus Evis Lucera 260 system and a BF-6C260 video-bronchoscope (Olympus Medical Systems, Tokyo, Japan)	[57]
Carcinoma *in situ*	Oral region	63 patients	Age: 57.9±16.7 (19-85) years Sex ratio: 1.9:1	ENF type V2 and type VQ NBI endoscopes (Olympus Medical Systems Corp., Tokyo, Japan)	[58]
Severe dysplasia, SCC, and other malignancies	Head and neck region	206 biopsies in 96 patients	Age: 66 ± 9.88 (35–86) years Sex ratio: 3.8:1	Olympus OTV-S7ProH-HD-12E HD autoclavable camera head, Olympus EVIS EXERA CLV-180 light source, 300 W xenon with narrow band imaging filter; Olympus EVIS EXERA CV-180 processor, high-definition television	[59]
Dysplasia/carcinoma *in situ*, microinvasive carcinoma	Oral cavity and oropharyngeal subsites	128 patients	Age: 65 (range 20-95) years Sex ratio: 0.7:1	Rigid telescope coupled to an Evis Exera II HDTV camera connected to an Evis Exera II CLV-180B light source (Olympus Medical Systems Corporation, Tokyo, Japan)	[61]
T1-T2 glottic cancers	Glottis	44 patients	Age: 54-83 years Sex ratio: 7.8:1	Video processor with integrated LED light source, model CV-170 with HD, ENF-VH, Olympus Corp	[66]
SCC	Oral cavity and oropharynx	16 patients	Age: 64.25 years Sex ratio: 2.2:1	Visera Elite system (OTV-S190 video processor and CLV-190 light source, OTV-S7Pro-10E HDTV camera; Olympus Medical Systems Corp, Tokyo, Japan) with rigid endoscopes	[67]
Carcinoma *in situ* and invasive SCC	Oral cavity, oropharynx, larynx, and hypopharynx	45 patients	Age: 62.8 (38-83) years Sex ratio: 4:1	Rigid video-endoscope connected to the Visera Pro CLV-S40Pro light source, HD Camera Head OTV-S7ProHHD-L08E, and Visera Pro OTV-S Pro image processor (Olympus Medical Systems, Tokyo, Japan)	[69]
Tis-T1a glottic cancer	Glottis	82 patients	Age: 67 (30-83) years Sex ratio: 5.8:1	Transnasal flexible videoendoscope (Olympus Medical System Corporation, Tokyo, Japan) with high-definition television (HDTV) white light (WL) and NBI	[72]
OSCC	Oral cavity	101 patients	Age: 52 (36-85) years Sex ratio: 3.7:1	Rhinolaryngovideoscope (ENF-V2), a light source (CLV-160B), and a video system center (CV-160B) (Olympus Medical Systems, Tokyo, Japan)	[74]
SCC and carcinoma *in situ*	Glottis, supraglottis, subglottis, and hypopharynx	56 patients	Age: 62.6 (42-83) years Sex ratio: 3.7:1	Evis Exera II optical system with CLV-180B light source (Olympus Medical Systems, Tokyo, Japan)	[75]
LSCC	Larynx	279 patients	Age: 65 years Sex ratio: 9.7:1	ENF-V2 videoendoscope connected to an Evis Exera II CLV-180B light source (Olympus Medical Systems Corporation, Tokyo, Japan)	[77]
Carcinoma *in situ*	Oral cavity, oropharynx, larynx, and hypopharynx	80 patients	Age: 20-70 years Sex ratio: 2.0:1	Not mentioned	[86]
Severe dysplasia and neoplasia	Tongue, buccal mucosa, gingiva, retro molar trigone, palate, palatoglossal arch, lower and upper fornix, floor of the mouth, labial commissure, and lip	91 patients	Age: 65.2±12.7 (29-98) years Sex ratio: 0.9:1	Visera Elite system (OTV-S190 video processor and CLV-190 light source, OTV-S7Pro-10E HDTV camera; Olympus Medical Systems Corp, Tokyo, Japan) with a rigid endoscope	[87]
Severe dysplasia and carcinoma	Larynx	138 patients	Age: 56 (37-84) years Sex ratio: 5.3:1	A magnifying endoscope (ENF-VT2; Olympus Optical Co., Tokyo, Japan) and a standard video endoscope system (VISERA Pro; Olympus)	[88]
Esophageal neoplasia	Esophagus	69 patients	Age: 53.9±10.38 years in patients with esophageal neoplasia, and 54.56±12.32 years in those without cancer lesions Sex ratio: 68:1	Evis Lucera Spectrum Video Imaging System (Olympus Optical Systems Co., Ltd., Tokyo, Japan)	[90]
Pharyngeal cancer	Hypopharynx	91 patients	Age: 64.9±7.9 years Sex ratio: 4.1:1	A magnifying endoscope with ×80 magnification (GIF-Q240Z; Olympus Optical, Tokyo, Japan)	[91]
HNSCC	Nasopharynx, oropharynx, larynx, hypopharynx, and oral cavity	59 patients	Not mentioned	ENF-V2 videoendoscope connected to the Evis Exera II CLV-180B light source (Olympus Medical Systems Corporation, Tokyo, Japan)	[93]
Sinonasal lymphoma, sinonasal malignant melanoma, and olfactory neuroblastoma	Nasal cavity	Case series of 4 patients	A 79-year-old female, a 50-year-old male, a 51-year-old female, and a 66-year-old male	Not mentioned	[94]

**Note** NBI, narrow band imaging; SCC, squamous cell carcinoma; OSCC, oral squamous cell carcinoma; LSCC, laryngeal squamous cell carcinoma; HNSCC, head and neck squamous cell cancer.

## LIST OF ABBREVIATIONS

NBI: Narrow band imaging
WLI: White light imaging
IPCLs: Intraepithelial papillary capillary loops
AFI: Autofluorescence imaging
CLE: Confocal laser endomicroscopy
SDSS: Sloan Digital Sky Survey
HPV: Human papillomavirus
CT: Computed tomography
MRI: Magnetic resonance imaging
OSCC: Oral squamous cell carcinoma
MVP: Microvascular hyperplasia pattern
COE: Conventional oral examination
TLM: Transoral laser microsurgery
SPM: Secondary primary malignancy
DOR: Diagnostic odds ratio
LSCC: Laryngeal squamous cell carcinoma
ESCC: Esophageal squamous cell carcinoma
AUC: Area under the curve
AI: Artificial intelligence
CNN: Convolutional neural network
